# Video-based physiologic monitoring: promising applications for the ICU and beyond

**DOI:** 10.1038/s41746-022-00575-z

**Published:** 2022-03-07

**Authors:** James A. Diao, Jayson S. Marwaha, Joseph C. Kvedar

**Affiliations:** 1 Program in Health Sciences and Technology, Harvard Medical School, Boston, MA USA; 2 Department of Surgery, Beth Israel Deaconess Medical Center, Boston, MA USA; 3 Department of Dermatology, Massachusetts General Hospital, Boston, MA USA

**Keywords:** Biomedical engineering, Respiratory signs and symptoms

## Abstract

The vital signs—temperature, heart rate, respiratory rate, and blood pressure—are indispensable in clinical decision-making. These metrics are widely used to identify physiologic decline and prompt investigation or intervention. Vital sign monitoring is particularly important in acute care settings, where patients are at higher risk and may require additional vigilance. Conventional contact-based devices, while widespread and generally reliable, can be inconvenient or disruptive to patients, families, and staff. Non-contact, video-based methods present a more flexible and information-dense alternative that may enable creative improvements to patient care. Still, these approaches are susceptible to several sources of bias and require rigorous clinical validation. A recent study by Jorge et al. demonstrates that video-based monitoring can reliably capture heart rate and respiratory rate and overcome many potential sources of bias in post-operative settings. This presents real-world evaluation of a practical, noninvasive, and continuous monitoring technology that had previously only been tested in controlled settings.

The modern hospital room is equipped with a battery of automated bedside monitors. The standard set—electrocardiography, impedance pneumography, and pulse oximetry—require probe attachments to multiple sites across the patient’s chest, fingers, or earlobe. While necessary, these sensors are often undesirable^1^. For patients, probes may irritate the skin, disrupt mobility and sleep, and harbor hospital-borne pathogens. For staff, wires and straps may interfere with patient transport, while inadvertent or purposeful sensor disconnections may require repeated attention, especially for delirious or agitated patients.

One proposed solution is video-based photoplethysmography, which extracts cardiorespiratory signals using light reflected from the skin. While this technology has been evaluated in diverse settings, including outpatient^2^, intraoperative^3^, and during hemodialysis^4^, most studies assessed brief, daytime measurements for healthy patients. To contribute evidence in less-controlled settings, Jorge et al.^5^ present data that video-based systems can deliver accurate, robust, and actionable vital sign estimates in the post-operative intensive care unit (ICU). The studied hardware consists of an optical camera, thermal camera, infrared light source, and privacy blind placed on a mobile trolley behind the foot of the bed. Captured video data were then processed for frame-by-frame changes attributable to cardiac and respiratory patterns^2^. Using this system, the authors observed 15 patients in the post-operative ICU for an average of 16 h each.

The resulting data support several important findings for video-based monitoring. First, the study demonstrated practical availability. After removing privacy periods (13% of total recorded time) and low-quality recording periods (43% for heart rate and 35% for respiratory rate), heart rate and respiratory rate were estimated for 44% and 51% of total recorded time, respectively. Vitals were measured regardless of patient position: lying in bed, sitting in a bedside chair, moving around, on ventilatory support, or receiving routine care. Of note, this study was the first to evaluate video-based monitoring overnight and for extended times (over 12 h) in the adult acute care setting. Second, video-based monitoring demonstrated reliable accuracy. When available, heart rate and respiratory rate were estimated with low mean absolute error (2.5 beats/min and 2.4 breaths/min) and low underestimation bias (−2.0 beats/min and −1.4 breaths/min) relative to reference values from contact-based methods. These metrics are consistent with previous studies on video-based monitoring and compare favorably to those of standard monitoring devices^2,4,6^.

Despite these reassuring results, much work remains for video-based methods to attain their full potential. Technical improvements may involve alternate sensors (e.g., multispectral, Doppler)^1^ or analysis methods (e.g., skin segmentation, deep learning)^7^. Beyond heart rate and respiratory rate, computational methods may also recover and magnify other signals with important health implications, including oxygen saturation, heart rate variability, volume status, distal perfusion, fall risk, activity, sleep, and delirium. The information density of visual inspection in routine clinical evaluation suggests that video-based methods may succeed for some of these applications and perhaps others not listed. For example, Jorge et al. retrospectively analyzed a recorded episode of diaphragmatic rupture with lung collapse and liver herniation, finding that the algorithm’s respiratory mapping aligned closely with known locations of thoracic and abdominal injury. Though further investigation is needed, this preliminary analysis suggests that video-based methods may capture more detailed physiologic information than conventional methods alone.

Future work should investigate the relative frequency of various failure modes (e.g., artifacts, signal loss, and false alarms), document their most common causes (e.g., patient motion or poor lighting), and demonstrate generalizability across sites and settings. In particular, replication in larger cohorts with improved demographic representation will be critical given potential inaccuracies in certain populations^8^, including patients with darker skin^9^. As physiologic surveillance technologies continue to improve, it will become increasingly important to address questions of bias, privacy, and possible misuse that inevitably arise with such tools.

The ubiquity of smartphones and webcam devices has dramatically expanded the feasible applications of video data in healthcare. Physiologic values that could previously only be measured using single-output, directly-attached probes in hospital settings may be increasingly derived using multichannel, no-contact monitoring devices serviceable in any environment. Such tools promise to improve care by characterizing longitudinal clinical status, alerting staff to detected emergencies, and capturing data from unwitnessed events for retrospective evaluation. Broader implementation may be fast approaching. During the COVID-19 pandemic, new regulatory guidance and reimbursement coverage has enabled rapid deployment of similar tools for remote patient monitoring^10^. Although technical, trust, and implementation challenges remain, we believe these may be overcome for a more robust, cost-effective, and unobtrusive future of medical data collection.
